# Supplementary material for: Multimodal Purcell enhancement and optical coherence of Eu3+ ions in a single nanoparticle coupled to a microcavity
Source: Nanophotonics. 2025 Feb 13;14(11):1817–26. doi: 10.1515/nanoph-2024-0721 (PMC12133218; doi:10.1515/nanoph-2024-0721)
Supplement: Supplementary file 1 — Supplementary Material Details [file j_nanoph-2024-0721_suppl_001.pdf]

Timon Eichhorn, Nicholas Jobbitt, Sören Bieling, Shuping Liu, Diana Serrano, Robert Huber, Tobias Krom, Ulrich Lemmer, Hugues de Riedmatten, Philippe Goldner, and David Hunger*

Supplementary Material for Multimodal Purcell enhancement and optical coherence of Eu^{3+} ions in a single nanoparticle coupled to a microcavity

1 Optical setup

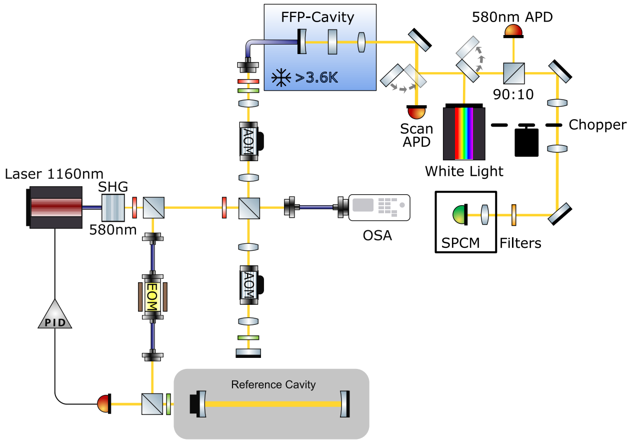

Fig. 1: Sketch of the optical setup used for cavity-enhanced spectroscopy of europium ions. PD: photodiode, SHG: second harmonic generation crystal, PID: PID-controller, EOM: electro-optic modulator, AOM: acousto-optic modulator, OSA: optical spectrum analyzer, APD: avalanche photodiode, SPCM: single photon counting module.

The setup used for the measurements presented in the paper is depicted in Fig. 1. The laser system consists of a 1160 nm external cavity diode laser (ECDL)(*DLpro*, Toptica Photonics AG) tuned by a digital laser controller (*DLCpro*, Toptica Photonics AG). The fiber-coupled laser output is guided into a temperature-stabilised (*TEC-1091 controller*, Meerstetter Engineering GmbH) second-harmonic-generation (SHG) stage (*WH-0580-000-A-B-C*, NTT Electronics, periodically poled lithium niobade (PPLN) waveguide), which outputs the frequency-doubled 580 nm laser light. To monitor the wavelength of our 580 nm laser module, some laser power is branched off into an optical spectrum analyzer (OSA)(*771A Laser Spectrum Analyzer*, Bristol Instruments). This device has a frequency resolution of 2 GHz and ±100 MHz accuracy in the visible domain.

The remaining laser power is guided into a 50 MHz-bandwidth acousto-optic modulator (AOM) (*AOMO 3200-121*, Gooch&Housego) in double-pass configuration in order to reach a six orders of magnitude suppression of the laser power for pulsed, resonant spectroscopy measurements. The light then passes another acousto-optic modulator (*AOMO 3350-199*, Gooch&Housego) with a higher bandwidth of 150 MHz in order to perform high-resolution sweeps of the laser frequency. In addition, this AOM is also used to create a two- or three-tone pattern to address the europium hyperfine levels. Finally, a quarter- and half-waveplate are placed in front of the fiber coupler to control the polarization of the excitation light. The fiber enters the cryostat via a vacuum-tight fiber feedthrough and is spliced to the cavity fiber(*SM530-125-160AL pure silica core*, ART Photonics GmbH) inside the cryostat.

The 1160 nm diode laser can be frequency stabilized to an external reference cavity to obtain a narrow laser linewidth for high resolution laser scans and to avoid frequency drifts over the long measurement times of several hours. We use the standard Pound-Drever-Hall stabilization technique [1] to lock the laser frequency to the narrow resonance of the reference cavity. A fiber-coupled electro-optic phase modulator(*PM594*, Jenoptik) is used to modulate sidebands with a 20 MHz frequency detuning. The cavity reflection signal is detected with a 50 MHz bandwidth APD(*APD120A/M*, Thorlabs GmbH). The demodulated, low pass filtered signal is fed into an FPGA-based feedback controller(*Digilock 110*, Toptica) which is connected to the laser piezo and current control to close the feedback loop. A laser frequency stability of about 20 kHz was measured when averaged over sev-

eral minutes timescale.

A motorized flip mirror(*MFF101/M*, Thorlabs GmbH) is placed behind the free-space output window of the cryostat to either guide the cavity transmission onto an APD(*APD120A/M*, Thorlabs GmbH) with 50 MHz bandwidth for cavity scans, or reflect the fluorescence light towards a single photon counting module. In order to stabilize the length of the microcavity to a particular resonance of the 580 nm laser, 10% of the power are coupled out onto another APD(*APD440A/M*, Thorlabs GmbH) which has a variable gain, a lower bandwidth of 10-100 kHz, but a higher sensitivity in order to achieve low intracavity power levels. Whenever just the 611 nm fluorescence of europium is detected, we can exchange the 90:10 beam splitter with a dichroic mirror(*MD588*, Thorlabs GmbH) to fully reflect the 580 nm transmission. The latter then serves as excitation and locking light simultaneously. The next element in the propagation direction is an optical chopper (*MC2000B-EC*, Thorlabs GmbH) where the chopper wheel is placed at the focus of a 4f-setup with focal length of 75 mm in order to minimize the transient time between the open and closed chopper windows. At a chopper frequency of 330 Hz, this results in a transient time of about 30 μs. The chopper is needed for pulsed measurements in order to block the excitation pulse, while the 580 nm fluorescence should be detected. We observed a higher dark count rate which exponentially decays over several milliseconds when the detector is gated on after an excitation pulse of a few nanowatts if no chopper is blocking the excitation pulse. We attribute this behaviour to heating of the sensor of the single photon counting module (SPCM)(*COUNT-100C*, LaserComponents). The SPCM is protected from ambient light with a light-tight box to reach the dark countrate of 20 cps. Additionally, a motorized filter positioner(*ELL9*, Thorlabs GmbH) is placed directly at the entrance hole of the box. Depending on the wavelength of the fluorescence light that we want to detect, it can be switched between a 580 nm band pass filter (*FF01-580/14-25*, Semrock), a combination of 610 nm band pass (*FF01-610/5-25*, Semrock) and 594 nm long pass (*BLP01-594R-25*, Semrock) filters or a 600 nm band pass filter (*84-785 BP 600/50*, Edmund Optics) with a 50 nm broad spectral window covering both fluorescence channels.

Lastly, it is also possible to insert a mirror on a magnetic mount into the collection optics beam path in order to shine in white light from a thermal lamp (*SLS201L/M*, Thorlabs GmbH) through the planar mirror and collect the cavity transmission through the fiber. The light filtered by the cavity is then guided to a Czerny-Turner spectrograph (*Shamrock 500i with iVAC316 LDC-DD camera*, Andor Technologies) via a fiber link and a spectrum of the white light cavity transmission can be taken. This is used to determine the cavity length from two resonances at wavelengths $\lambda_{1,2}$ differing by one FSR according to:

$$d_c = \frac{\lambda_1 \lambda_2}{2(\lambda_1 - \lambda_2)}. \quad (1)$$

2 Cavity Transmission Scan

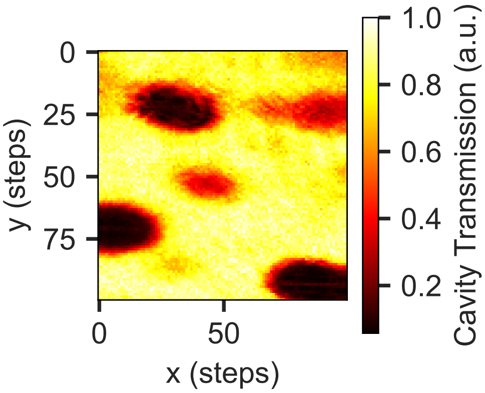

Fig. 2: Cavity transmission scan of several NPs of different sizes. Since the diameters of the scatterers are much smaller than the cavity mode diameter of about 3 μm, the cavity mode profile is visible on a scatterer. All NPs except the right-bottom one show the fundamental TEM_{00} mode. The right-bottom NP has a larger diameter and thus shows a TEM_{10} mode which gives the highest transmission on this NP since this mode is less affected by the scattering losses compared to the TEM_{00} mode.

3 Inhomogeneous lines

As a reference, the width of the inhomogeneous line of a powder of 60 nm Eu^{3+}:Y_2O_3 nanoparticles was measured by photoluminescence excitation laser spectroscopy inside a cryostat at 1.4 K at Chimie ParisTech. The data together with a fit of a Lorentzian line can be seen in Fig. 3. The fit reveals an inhomo-

geneous linewidth of 34.49(0.01) GHz (FWHM). This value is taken as an ensemble-averaged reference for the inhomogeneous lines we measured inside the cavity on six different individual nanoparticles as depicted in Fig. 4A-F.

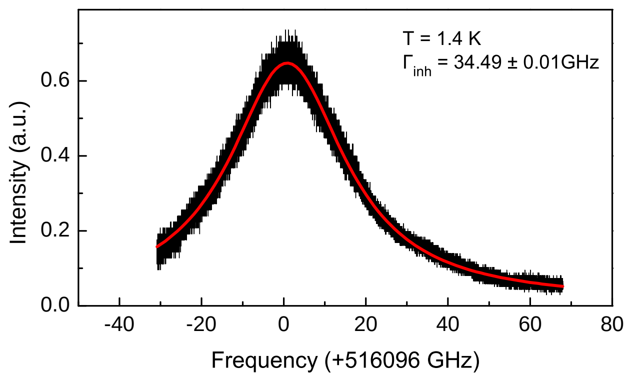

Fig. 3: Photoluminescence excitation laser scan over the inhomogeneous distribution of europium ions of a powder of 60 nm yttria NPs of 0.3% doping concentration at 1.4 K.

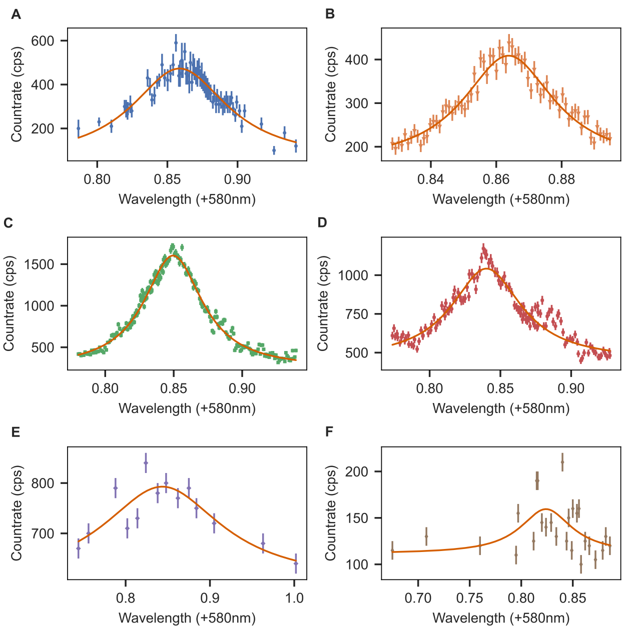

Fig. 4: PLE scans of the inhomogeneous line of europium ions for six different nanoparticles at 20 K. A Lorentzian line (red) is fit to the data to extract the full width at half maximum (FWHM).

4 Lifetime histograms

In order to experimentally determine the effective Purcell factors for the different NPs, we measure the free-space and three different cavity-enhanced lifetimes (only 580 nm transition, only 611 nm transition and both simultaneously) of the six different nanoparticles, shown in Fig. 5A-F. Each photon count histogram is first fit with a mono-exponential decay curve to extract the background counts. Thereafter, this background value is subtracted from the data to see a linear decay curve on a semi-logarithmic plot. Finally, a linear function is fit to the normalized, background-subtracted logarithmic histogram to reveal the lifetime as the inverse slope of the line. The error on the lifetime is set to 0.1 ms due to deviations found when subtracting different background values. Since the measurement method for the multimodal Purcell-enhancement evolved over the course of these measurements, we didn't measure all four histograms for each NPs.

5 Derivation of the ensemble average Purcell factor

The effective Purcell factor $F_{\mathrm{P}}^{\mathrm{eff}}$, given in the paper is reduced by three main correction factors in a realistic Fabry-Pérot cavity, namely:

1. The cavity length jitter correction $\xi_{\mathrm{vibrations}}$.
2. The dipole orientation correction ξ_{dipole}.
3. The electric field correction ξ_{field}.

The maximum and ensemble averaged effective Purcell factors can thus be described as

$$F_{\mathrm{P}}^{\star}(d_{\mathrm{NP}}) = \xi_{\mathrm{field}}(d_{\mathrm{NP}})\,\xi_{\mathrm{vibrations}}\,F_{\mathrm{P}}^{\mathrm{eff}}(d_{\mathrm{NP}}),$$
$$\langle F_{\mathrm{P}}^{\star}(d_{\mathrm{NP}})\rangle = \langle \xi_{\mathrm{field}}(d_{\mathrm{NP}})\rangle\,\langle \xi_{\mathrm{dipole}}\rangle\,\xi_{\mathrm{vibrations}}\,F_{\mathrm{P}}^{\mathrm{eff}}(d_{\mathrm{NP}}).$$

The dependence on the nanoparticle diameter d_{NP} is written explicitly, since it has a strong influence on the cavity finesse which enters the Purcell factor according to Eq. 1 in the paper. Quantities in angled brackets denote an average value for a large ensemble of ions inside a single nanoparticle.

First, the cavity length jitter around the peak of the resonance, as anaylzed in detail in [2], leads to a correction factor [3]

$$\xi_{\mathrm{vibrations}} = \sqrt{\frac{\pi}{8}}\frac{\delta d}{\sigma_z} e^{\frac{\delta d^2}{8\sigma_z^2}} \left(1 - \mathrm{erf}\left(\frac{\delta d}{2\sqrt{2}\sigma_z}\right)\right) = 0.63,$$

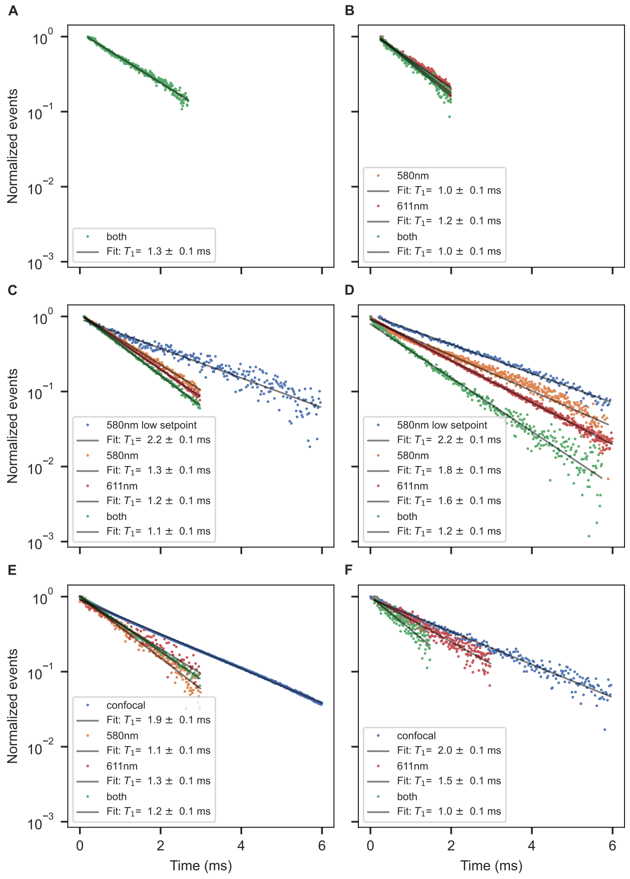

Fig. 5: Cavity-enhanced (orange, red, green) and free-space (blue) lifetime measurements of six different NPs. The different colors indicate the Purcell-enhanced transition(s): orange: only 580 nm transition, red: only 611 nm transition and green: both transitions are resonant simultaneously. The black lines are linear fits to the normalized and background-corrected logarithmic data to extract the lifetime T_1.

which is the same for every emitter inside the nanoparticle. A Gaussian linewidth broadening of standard deviation $\sigma_z = 8\,\mathrm{pm}$ is assumed, which represents the cavity stability during most of the lifetime measurements. The FWHM cavity linewidth is calculated as $\delta d = 14.5\,\mathrm{pm}$.

Secondly, the overlap between the transition dipole and the electric field vector is set to one for the maximum Purcell factor. However, in general, the dipole orientation is random due to the polycrystalline structure of the nanoparticles and three different possible transition dipole orientations of an europium ion at the C_2 site in yttria [4]. Thus, an ensemble average over all possible orientations has to be taken. The Purcell factor is proportional to the squared dot product of both vectors, which is expressed in spherical coordinates,

$$F_\mathrm{P}^\star(\theta,\varphi) = F_\mathrm{P}\sin^2\theta\,\cos^2\varphi.$$

The ensemble averaging is done analytically by the integration over the 1st octant of the unit sphere, assuming a uniform distribution of orientations and an electric field vector lying on the x-axis, i.e. $\vec{e}_d\cdot\vec{e}_E = \sin\theta\,\cos\phi$:

$$\langle\xi_\mathrm{dipole}\rangle = \frac{\langle|\vec{d}\cdot\vec{E}_{max}|^2\rangle}{|\vec{d}|^2|\vec{E}_{max}|^2} = \\ = \frac{1}{N(\theta,\phi)}\int_0^{\frac{\pi}{2}}\int_0^{\frac{\pi}{2}}\sin^2\theta\,\cos^2\phi\,\sin\theta\,\mathrm{d}\theta\,\mathrm{d}\phi = \\ = \frac{1}{N(\theta,\phi)}\frac{\pi}{6} = \frac{1}{3},$$

with the normalization factor $N(\theta,\phi) = \int_0^{\frac{\pi}{2}}\int_0^{\frac{\pi}{2}}\sin\theta\,\mathrm{d}\theta\,\mathrm{d}\phi = \pi/2$.

Lastly, we take into account the random position of europium ions inside the nanoparticle, which results in a random position in the standing wave field of the cavity mode. The lateral extent of the nanoparticle is much smaller than the cavity mode waist, such that we assume every ion to be placed at the lateral field maximum of the Gaussian cavity mode. The DBR mirror coating has a spacer layer, such that the field maximum is located $z_\mathrm{max} = 40\,\mathrm{nm}$ above the mirror surface. The Purcell factor is hence reduced depending on the z-position of the emitter according to:

$$F_\mathrm{P}^\star(z) = F_\mathrm{P}\cos^2\left(\frac{2\pi}{\lambda}(z - z_\mathrm{max})\right).$$

The field correction factor for a maximally coupling emitter is then defined as as,

$$\xi_\mathrm{field}(d_\mathrm{NP}) = \begin{cases}\cos^2\left(\frac{2\pi}{\lambda}(z - z_\mathrm{max})\right) & \text{for } d_\mathrm{NP} < z_\mathrm{max},\\ 1 & \text{for } d_\mathrm{NP} \geq z_\mathrm{max}.\end{cases}$$

The ensemble averaged field correction factor $\langle\xi_\mathrm{field}\rangle$ is calculated by integration over all z positions within the nanoparticle, assuming a uniform density of ions, i.e. a cube-shaped nanoparticle for ease of calculation.

$$\langle\xi_\mathrm{field}(d_\mathrm{NP})\rangle = \left[\frac{1}{d_\mathrm{NP}}\int_{z=0}^{d_\mathrm{NP}}\cos\left(\frac{2\pi}{\lambda}(z - z_\mathrm{max})\right)\mathrm{d}\,z\right]^2 \\ = \left(\frac{\lambda}{2\pi d_\mathrm{NP}}\right)^2\cdot \\ \cdot\left[\sin\left(\frac{\lambda}{2\pi}(d_\mathrm{NP} - z_\mathrm{max})\right) + \sin\left(\frac{\lambda}{2\pi}z_\mathrm{max}\right)\right]^2.$$

In principle, the effective Purcell factor can also be further reduced by the quantum efficiency, QE, of the emitter,

$$F_{\mathrm{P}}^{\mathrm{eff}} = QE \cdot \zeta \cdot F_{\mathrm{P}},$$

which is defined as the ratio $QE = \gamma_r/\gamma_{tot}$ of the radiative decay rate γ_r by the total decay rate $\gamma_{tot} = \gamma_r + \gamma_{nr}$, also including non-radiative decay channels e.g. via phonons. However, according to measurements of Buijs et al. [5], non-radiative quenching of the $^5D_0$ state at impurities of the crystal lattice is negligible for doping concentrations below 10%. Thus, for our sample with 0.3% doping, we can assume a near-unity quantum efficiency and thus neglect it in the following.

6 Multimodal Purcell factor

The generalized Purcell factor can be defined using Fermi's golden rule:

$$F_P = \frac{1}{\gamma_0}\frac{2\pi}{\hbar^2}\int_0^\infty M_0^2(\omega)\Lambda(\omega)\rho(\omega)\,\mathrm{d}\omega.$$

Here, $\Lambda(\omega)$ and $\rho(\omega)$ denote the density of states of the emitter and cavity, respectively, and are Lorentzian lines in our case. For our case of two far-detuned $|\omega_{580\mathrm{nm}} - \omega_{611\mathrm{nm}}| \gg \Gamma_{580/611\mathrm{nm}}, \kappa_{580\mathrm{nm}/611\mathrm{nm}}$ emitter and cavity resonances, there is no cross spectral overlap between the 580 nm cavity and 611 nm emitter modes and vice versa. Therefore, the cross-terms in the above equation vanish:

$$\int_0^\infty M_{580\mathrm{nm}}^2(\omega)\,\Lambda_{580\mathrm{nm}}(\omega)\,\rho_{611\mathrm{nm}}(\omega)\,\mathrm{d}\omega \approx \int_0^\infty M_{611\mathrm{nm}}^2(\omega)\,\Lambda_{611\mathrm{nm}}(\omega)\,\rho_{580\mathrm{nm}}(\omega)\,\mathrm{d}\omega \approx 0.$$

This results then in a linear sum of the two Purcell factors as given in the manuscript:

$$F_{P,\mathrm{both}} = F_{P,580\mathrm{nm}} + F_{P,611\mathrm{nm}}.$$

7 Coherence properties of a powder of 60 nm NPs

We also performed photon echo (PE) measurements on a powder of the 60 nm yttria NPs with 0.3% europium doping concentration as we used for the measurements in the paper. This spectroscopy technique reveals the coherence time of the emitters, and details on the measurement can be found in [6]. A PE measurement at cryogenic temperatures of 1.4 K taken at Chimie ParisTech can be seen in Fig. 6, together with an exponential fit to extract a coherence time of $T_2 = 2.74(0.05)\,\mu\mathrm{s}$, corresponding to a homogeneous linewidth of 116(2) kHz.

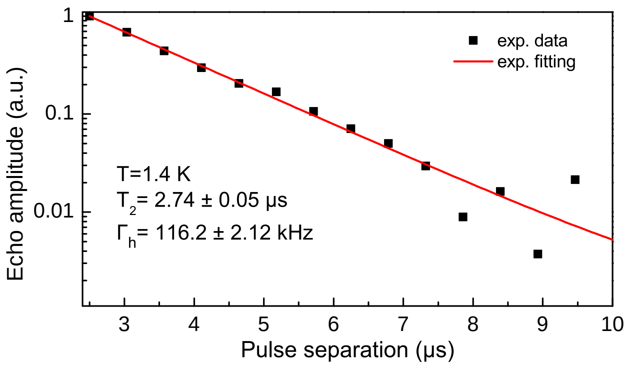

Fig. 6: Photon echo measurement of a powder of 60 nm yttria NPs of 0.3% doping concentration at 1.4 K.

References

[1] R. W. P. Drever, J. L. Hall, F. V. Kowalski, J. Hough, G. M. Ford, A. J. Munley, and H. Ward, "Laser phase and frequency stabilization using an optical resonator," *Applied Physics B Photophysics and Laser Chemistry*, vol. 31, pp. 97–105, June 1983.

[2] M. Pallmann, T. Eichhorn, J. Benedikter, B. Casabone, T. Hümmer, and D. Hunger, "A highly stable and fully tunable open microcavity platform at cryogenic temperatures," *APL Photonics*, vol. 8, p. 046107, Apr. 2023.

[3] Y. Fontana, R. Zifkin, E. Janitz, C. D. Rodríguez Rosenblueth, and L. Childress, "A mechanically stable and tunable cryogenic Fabry–Pérot microcavity," *Review of Scientific Instruments*, vol. 92, p. 053906, May 2021.

[4] A. Fossati, S. Liu, J. Karlsson, A. Ikesue, A. Tallaire, A. Ferrier, D. Serrano, and P. Goldner, "A Frequency-Multiplexed Coherent Electro-optic Memory in Rare Earth Doped Nanoparticles," *Nano Letters*, vol. 20, pp. 7087–7093, Oct. 2020.

[5] M. Buijs, A. Meyerink, and G. Blasse, "Energy transfer between Eu3+ ions in a lattice with two different crystallographic sites: Y2O3:Eu3+, Gd2O3:Eu3+ and Eu2O3," *Journal of Luminescence*, vol. 37, pp. 9–20, Apr. 1987.

[6] A. Fossati, D. Serrano, S. Liu, A. Tallaire, A. Ferrier, and P. Goldner, "Optical line broadening mechanisms in rare-earth

doped oxide nanocrystals," *Journal of Luminescence*, vol. 263, p. 120050, Nov. 2023.
